# Liver fibrosis and type 2 diabetes modulate postprandial incretin and glucagon responses in fatty liver disease

**DOI:** 10.1007/s13105-026-01141-x

**Published:** 2026-02-06

**Authors:** Brenno Astiarraga, Adrià Rodriguez-Castellano, Victoria Ceperuelo-Mallafré, Anna Marsal-Beltran, Francisco J. Osuna-Prieto, Nerea Vilanova, Jordi Gracia-Sancho, Joan Carles Quer, Ana Megía, Albert Pardo Balteiro, Joan Vendrell, Sonia Fernández-Veledo

**Affiliations:** 1 Institut d’investigació Sanitària Pere Virgili (IISPV), 43005 Tarragona, Spain; 2 CIBER de Diabetes y Enfermedades Metabólicas Asociadas (CIBERDEM), Instituto de Salud Carlos III, Madrid, Spain; 3 Universitat Rovira I Virgili (URV), 43201 Reus, Spain; 4 Liver Vascular Biology Lab, IDIBAPS – Hospital Clínic Barcelona – CIBEREHD, 08036 Barcelona, Spain; 5 Servicio de Aparato Digestivo, Hospital Universitari Joan XXIII, 43005 Tarragona, Spain; 6 Servicio de Endocrinología y Nutrición, Hospital Universitari Joan XXIII, 43005 Tarragona, Spain

**Keywords:** Glucagon, GLP-1, GLP-2, GIP, Metabolic-associated steatotic liver disease, Liver fibrosis, Type 2 diabetes

## Abstract

The study aims to characterize the secretion dynamics of glucagon-related peptides, including GLP-1, GIP, and GLP-2, across different stages of metabolic-associated steatotic liver disease (MASLD), while evaluating the impact of type 2 diabetes (T2D) on these hormonal responses. Thirty-four MASLD subjects were stratified according with the liver transient elastography (TE ≥ 9 kPa) and T2D in NF (no fibrosis, without T2D; *n* = 12), NFD (no fibrosis, with T2D; *n* = 8), F (fibrosis, without T2D; *n* = 5), and FD (fibrosis, with T2D; *n* = 9) and completed a standardized 3-h meal tolerance test (MTT). The presence of liver fibrosis, regardless of diabetes status, was associated with hyperglycemia, hyperinsulinemia, and greater insulin resistance compared to the non-fibrosis (NF) group. Significant differences in glucagon and GLP-1 response curves were observed across groups. People with T2D showed an elevated peak of glucagon and increased glucagon exposure, as indicated by both the 60-min area under the curve (AUC60’) and total AUC during the MTT. In the FD group, fasting and peak GLP-1 levels, as well as AUC60’ and total AUC GLP-1, were 1.9-, 1.8-, and 1.9-fold higher, respectively, compared to the NF group. GIP responses were similar across groups, except for elevated fasting levels in NFD (*p* = 0.002). GLP-2 mirrored GLP-1, with FD showing the highest fasting and postprandial levels. Stepwise regression identified fibrosis and FPG as the main predictors of GLP-1, while glucagon was linked to FPG, HbA1c, and BMI. Liver fibrosis and T2D impact glucagon-related peptides responses in MASLD, revealing important metabolic alterations that may guide therapeutic approaches.

## Introduction

Metabolically associated steatotic liver disease (MASLD), formerly known as non-alcoholic fatty liver disease, encompasses a spectrum of disorders ranging from benign steatosis to metabolic dysfunction-associated steatohepatitis (MASH), liver fibrosis, cirrhosis, and hepatocellular carcinoma. These conditions are often linked to metabolic disorders such as obesity and type 2 diabetes (T2D) [1]. The etiology and pathophysiology of MASLD are influenced by a complex interplay of genetic, metabolic, and environmental factors [1, 2]. T2D plays a significant role in the progression of MASLD by impairing insulin action in the liver [2, 3], adipose tissue, and skeletal muscle, leading to hepatic triglyceride accumulation [4]. This, in turn, triggers mitochondrial dysfunction, oxidative stress, and hepatocellular apoptosis in the liver, ultimately contributing to fibrosis [5–7]. Notably, MASLD itself can increase the risk of developing T2D, highlighting the need for screening for both conditions [3, 8]. The bidirectional relationship between MASLD and T2D complicates disease management by worsening insulin resistance and pancreatic beta-cell function [8].

Glucagon plays a crucial role in regulating glucose homeostasis, primarily by influencing glucose production, contributing to energy availability, and modulating insulin release [9, 10]. Both fasting and postprandial glucagon levels are often disrupted in individuals with prediabetes and T2D [9, 10]. Similarly, elevated glucagon levels have been reported in patients with MASLD and MASH [11, 12]. However, whether these elevated glucagon levels are associated with markers of liver fibrosis remains unknown [13].

Among the most physiologically significant glucagon-related peptides, the incretin hormones glucagon-like peptide-1 (GLP-1) and glucose-dependent insulinotropic polypeptide (GIP) play a crucial role in glucose homeostasis. These peptides substantially potentiate glucose-stimulated insulin secretion, accounting for approximately 40–60% of postprandial insulin release. This incretin effect is diminished in both obesity and glucose intolerance [14]. While GLP-1 secretion is reduced in MASLD and MASH, GIP secretion remains intact [15]. Importantly, GLP-1 not only stimulates insulin secretion but also suppresses glucagon release during hyperglycaemia [16]. By contrast, GIP, even in T2D, paradoxically stimulates glucagon secretion, potentially contributing to disease progression [16]. Glucagon-like peptide-2 (GLP-2), structurally related to glucagon but functionally distinct, is co-secreted with GLP-1 from enteroendocrine L-cells. Although it lacks incretin activity, GLP-2 plays a vital role in intestinal homeostasis by promoting intestinal growth, enhancing nutrient absorption, and maintaining gut barrier integrity [17]. These functions indirectly contribute to liver health by modulating the gut-liver axis [17, 18]. Low-dose GLP-2 therapy has been shown to ameliorate hepatic steatosis in a rat model of MASLD with short bowel syndrome [19]. The dual GLP-1/GLP-2 receptor agonist, dapiglutide, offers additional benefits by reducing gut permeability, exerting anti-inflammatory effects in the intestine, and facilitating liver regeneration through modulation of the microbiome and the gut-liver axis [20].

The severity of MASLD, particularly in the context of liver fibrosis and T2D, may influence the secretion dynamics of glucagon-related peptides in response to nutrient intake. We hypothesize that liver fibrosis and T2D could independently or synergistically alter fasting and postprandial incretin responses, potentially reflecting adaptive mechanisms to hepatic metabolic stress. By investigating these hormonal profiles across MASLD stages, this study aims to uncover how fibrosis and diabetes interact to modulate enterohepatic communication, providing insights into MASLD pathophysiology and identifying potential therapeutic targets for disease management.

## Methods

### Population

This single-center observational study was designed to investigate fasting levels and postprandial responses of glucagon-related peptides (glucagon, GLP-1, GLP-2, and GIP) in patients with MASLD, stratified by T2D status and liver fibrosis. The study cohort comprised 34 individuals referred to the hepatology outpatient clinic at Joan XXIII University Hospital (Tarragona, Spain) between 2019 and 2023. MASLD diagnosis was established based on the presence of cardiometabolic risk factors and/or hepatic steatosis detected by imaging techniques (primarily ultrasound), combined with a Fatty Liver Index (FLI) ≥ 60, in the absence of high-risk alcohol consumption or other concomitant liver diseases. This diagnostic approach aligns with the recommendations outlined by Caballeria et al. (2019) for the detection, diagnosis, and follow-up of patients with non-alcoholic fatty liver disease in primary and hospital care settings [21]. Inclusion criteria were age ≥ 18 years and hepatic steatosis estimated by FLI index (≥ 60), with and without T2D. Exclusion criteria included pregnancy, known liver diseases of other aetiology (e.g., viral hepatitis B/C infection, autoimmune liver disease, drug-related liver disease), or significant alcohol consumption (> 21 units/week for males or > 14 units/week for females), and insulin treatment.

Initial screening involved collecting demographic information, comorbidities, and medication history. Hepatic steatosis was assessed using the FLI index (≥ 60), and liver stiffness was assessed using transient elastography (TE), with a threshold of ≥ 9 kPa used to estimate liver fibrosis [22]. T2D diagnosis was based on a fasting plasma glucose (FPG) level ≥ 7.0 mmol/L, an HbA_1c_ level ≥ 48 mmol/mol, or a previous clinical diagnosis [23]. Among participants in the NFD and FD groups (defined below), 25% and 44%, respectively, were receiving hypolipidaemic treatment, while 63% and 67%, respectively, were receiving hypoglycaemic treatment. Patients were instructed to discontinue hypoglycemic medications 24 h before the test. Those treated with GLP-1 receptor agonists suspended the medication at their previous dose before the study.

Eligible patients were divided into four groups based on the criteria mentioned above: non-fibrosis group without T2D (NF group, *n* = 12), non-fibrosis group with T2D (NFD group, *n* = 8), fibrosis group without T2D (F group, *n* = 5), and fibrosis group with T2D (FD group, *n* = 9). The NF group served as the comparator to evaluate differences across the other groups. The detailed characteristics of the participants are shown in Table 1**.** The study was conducted in accordance with the Declaration of Helsinki. It was approved by the Ethical Committee for Drugs Research of the Pere Virgili Institute for Health Research–IISPV, Tarragona, Spain (reference number: 151/2019). All the patients provided written informed consent before participating.

**Table 1:** Anthropometric and clinical characteristics of the study participants

variables	NF	NFD	*p*1	F	FD	*p2*	ANOVA
Age (years)	55 [18]	58 [12]	0.314	58 [11]	59 [8]	1.000	0.144
Gender (F/M)	4/8	2/6	––	1/4	6/3	––	––
BMI (kg/m^2^)	30.2 [6.4]	30.3 [3.1]	0.964	30.4 [8.3]	30.1 [12.7]	0.92	0.63
Fat mass (%)	30.9 ± 7.9	32.6 ± 5.2	0.895	35.7 ± 7.7	36.5 ± 7.3	0.999	0.312
Waist (cm)	103.2 ± 12.7	107.3 ± 6.8	0.838	110.0 ± 15.5	110.1 ± 4.4	1.000	0.57
FLI	87 [13]	91 [11]	0.989	92 [22]	88 [18]	0.997	0.956
ET (pka)	5.1 [3.0]	6.0 [2.0]	1.000	9.8 [12.0] ^**^	13.8 [8.0] ^****^	0.872	0.0001
Glucose (mmol/l)	5.2 [0.8]	8.6 [2.1]	0.0001	5.6 [0.9]	7.2 [2.1] ^***^	0.01	0.0001
Insulin (pmol/l)	94.0 ± 54.3	119.9 ± 38.2	0.489	123.4 ± 93.6	145.7 ± 58.3	0.823	0.187
HbA_1c_ mmol/mol	37.7 [2.8]	50.3 [11.7]	0.0001	38.3 [4.9]	49.7 [12.0] ^**^	0.035	0.001
Urate (mg/dl)	6.3 [2.9]	6.4 [3.4]	0.968	5.2 [3.8]	5.0 [2.5]	0.958	0.388
Urea (mg/dl)	36.0 [12.0]	45.4 [18.2]	0.131	34.4 [27.0]	34.0 [7.1]	0.887	0.097
Creatine (mg/dl)	0.87 [0.21]	0.86 [0.24]	0.839	0.77 [0.31]	0.67 [0.18]	0.413	0.043
T. Cholesterol (mmol/l)	5.1 ± 0.8	4.1 ± 0.9	0.143	5.5 ± 1.5	4.3 ± 1.0	0.287	0.061
HDLc (mmol/l)	1.2 [0.3]	1.0 [0.3]	0.132	2.0 [1.2]	1.1 [0.5]	0.127	0.015
LDLc (mmol/l)	2.9 ± 0.9	2.2 ± 1.3	0.206	3.2 ± 1.6	2.4 ± 0.9	0.332	0.094
Triglycerides (mmol/l)	1.3 [1.4]	1.7 [1.5]	0.944	0.9 [0.7]	1.6 [0.5]	0.415	0.26
AST (U/L)	31.5 [13.8]	22.5 [8.5]	0.454	52.0 [168.5] ^*^	37.0 [20.5]	0.131	0.002
ALT (U/I)	40.5 [22.3]	35.0 [18.8]	0.805	65.0 [179.0]	49.0 [19.5]	0.586	0.074
GGT (U/L)	57.0 [83.8]	57.0 [112.0]	0.994	199.0 [625.5] ^*^	55.0 [62.0]	0.017	0.012
Hypolipemiants (%)	0	25	––	0	44	––	––
Oral Hypoglycemic medications (%)	0	63	––	0	67	––	––
GLP-1 therapy (%)	0	25	––	0	33	––	––
SGLT-2 inhibitors (%)	0	50	––	0	11	––	––

Data are mean ± SD or median [IQR] depending on data distribution. Group differences were assessed by one-way ANOVA with Tukey’s post-hoc test for pairwise comparisons. *p*1 indicates the comparison between NF and NFD groups, while *p*2 shows the comparison between F and FD groups. The symbol (*) indicates the *p-*values for the comparison between both liver fibrosis groups (F and FD) and NF group (reference group) as follows: *p < 0.05, **p < 0.01, ***p < 0.001, and ****p < 0.0001. Abbreviations: Fatty liver index (FLI), transient elastography (ET), aspartate aminotransferase (AST), alanine aminotransferase (ALT), gamma-glutamyl transpeptidase (GGT). Medication use is reported as the percentage of patients receiving each treatment

### Body composition and metabolic testing

Height, weight, and waist circumference were assessed during the study visit. Body composition was estimated using bioelectrical impedance analysis (Tanita Europe BV, Amsterdam, The Netherlands). After a 10–14 h fast, all participants underwent a 3-h meal tolerance test (MTT) between 08:00–9:00 AM. During the MTT, participants consumed 300 ml of Isosource® Energy (478 kcal, 16% protein, 49% carbohydrates, 30% lipids; Nestlé Health Science, Lausanne, Switzerland) and remained reclined on a medical bed. An intravenous catheter was inserted into the antecubital vein and maintained at 50 °C with a thermal pad. After a 30-min rest period, the test was started. Blood samples were collected at baseline (−30 and 0 min before the meal) and at 10, 20, 30, 60, 90, 120, 150, and 180 min post-meal.

### Determinations

For serum samples, blood was allowed to clot for 30 min at room temperature, then placed on ice and centrifuged. Plasma samples intended for incretin and glucagon analysis were protected from enzymatic degradation by adding protease inhibitors (20–201 and/or DPP4-010; EMD Millipore Corp., St. Louis, MO). These samples were immediately chilled on ice until centrifugation, then aliquoted and stored at −80 °C until analysis.

Plasma lipid, hepatic, and renal profiles, and HbA_1c_ levels were determined using standard laboratory methods. Bedside plasma glucose was determined by the glucose oxidase method (GM9 analyser; Analox, London, UK). Plasma insulin and C-peptide levels were quantified by immunochemiluminometric assay (ADVIA Centaur; Siemens Healthineers, Erlangen, Germany). Plasma total GLP-1, GLP-2, GIP, and glucagon levels were measured by ELISA kits (EZGLP1T-36 K, EZGLP2T-37 K, and EZHGIP-54 K from Merck KGaa, Darmstadt, Germany, and 10–1271-01 from Mercodia, Uppsala, Sweden). The intra- and inter-assay coefficients of variation were as follows: GIP, 4.6% and 6.4%; GLP-1, 5.4% and 5.0%; GLP-2, 1.9% and 11.9%; and glucagon, 4.9% and 15.4%, respectively. The assays for insulin and C-peptide demonstrated coefficients of variation below 5%, consistent with the laboratory quality standards.

### Calculations

Fasting insulin resistance was estimated using the HOMA-IR index. Whole-body insulin resistance during the MTT was assessed using the insulin sensitivity index (ISI-Matsuda). Both indices were calculated using a publicly available online calculator (http://mmatsuda.diabetes-smc.jp/englishsi.html) with corrections for glucose dose as recommended. The AUC was computed using the trapezoidal rule, with specific calculations for the first hour of the test (AUC60') and for the entire test (total AUC), and expressed as the mean value per minute during the relative period. The incremental AUC (iAUC) was obtained by subtracting the relative fasting value area from the partial (60 min) or total AUC. Early pancreatic beta-cell responses were assessed using the insulinogenic index (IGI). Additionally, the total insulin response (TIR) during the MTT was estimated as the ratio of the total AUC of insulin or C-peptide to the AUC of glucose.

## Statistical analysis

Data following a normal distribution are presented as mean ± SD. Non-parametric data, determined by the Shapiro–Wilk normality test, are presented as median and [IQR]. Non-parametric data were log_10_-transformed before analysis. One-way ANOVA was used to compare group differences, with Tukey’s post-hoc tests for pairwise comparisons. ANOVA for repeated measures was used to evaluate response kinetics during the MTT. Unless otherwise indicated, the *p-*value for the interaction (p_int_) between group*treatment is reported in the text. Pairwise group comparisons were conducted when appropriate. A linear stepwise regression was employed to examine the impact of liver steatosis (FLI index), liver fibrosis (TE value), body composition (BMI), and glucose tolerance (FPG or HbA_1c_) on the variables of interest. Outliers were identified and excluded using standard descriptive analysis due to data variability. Statistical analyses were performed using IBM SPSS Statistics version 19 (IBM Corp., Armonk, NY). Figures were generated using GraphPad 8 (GraphPad Software Inc., Boston, MA).

## Results

### Fibrosis disrupts glucose homeostasis and insulin sensitivity in patients with MASLD without T2D

**Fig. 1:**
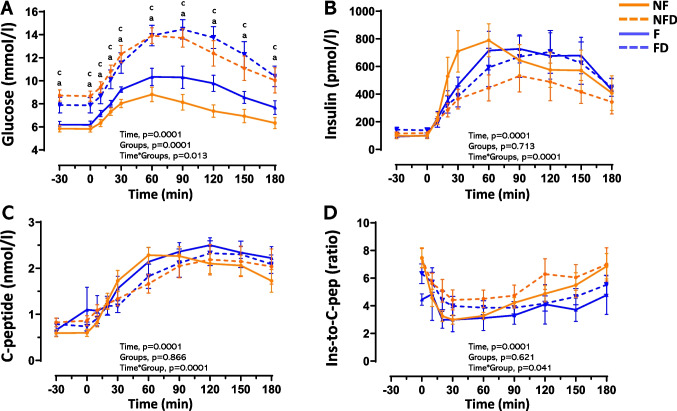
Glucose, insulin, and C-peptide dynamics during MTT in patients with MASLD. Panels A–C show glucose, insulin, and C-peptide profiles, respectively, and panel D the insulin-to-C-peptide ratio. Curves-profiles were analysed by repeated-measures ANOVA while one-way ANOVA was used to explore time point differences. Tukey post-hoc results (a, b, c) indicate differences (*p* < 0.05) between NFD, F, and FD groups vs. the NF group, respectively. Abbreviations: NF, no liver fibrosis or T2D; NFD, no liver fibrosis with T2D; F, liver fibrosis without T2D; FD, liver fibrosis with T2D

Patients were categorised into four groups based on their liver fibrosis and T2D status (Table 1). Groups were similar for age, sex, body composition, and FLI index. Both fibrosis groups (F and FD) exhibited similarly elevated liver stiffness, irrespective of T2D status. As expected, people with T2D exhibited elevated fasting and postprandial glucose response compared to the NF group (*p*_*int*_ = 0.013; Fig. 1A). Notably, the group with fibrosis alone (F group), while showing no changes in fasting glucose levels, exhibited an intermediate glucose response to the MTT. Specifically, glycemia at 120 min was significantly higher in the F group (9.8 ± 1.6 mmol/l) than in the NF group (7.4 ± 1.9 mmol/l) (*p* = 0.027), and the AUC for glucose was 1.2 times higher (*p* = 0.014) (Fig. 1A).

The insulin response varied significantly across groups (p_int_ = 0.0001) (Fig. 1B). The NF group exhibited an insulin response that most closely resembled a physiological pattern, with an initial insulin increase followed by a gradual decline throughout the test. In contrast, the NFD and FD groups displayed a markedly impaired response, characterized by a nearly absent early insulin peak and sustained elevated insulin levels during the test. Notably, the F group exhibited an insulin response pattern similar in timing to that observed in people with T2D, regardless of whether fibrosis was present. This suggests that the temporal profile of insulin secretion in the F group aligns with the characteristic response seen in individuals with T2D, despite the absence of a diabetes diagnosis (Fig. 1B). Similar trends were observed for C-peptide levels (Fig. 1C). Hepatic insulin clearance, assessed as the ratio of circulating insulin to C-peptide concentrations, also differed significantly across groups (*p*_int_ = 0.041; Fig. 1D).

**Fig. 2:**
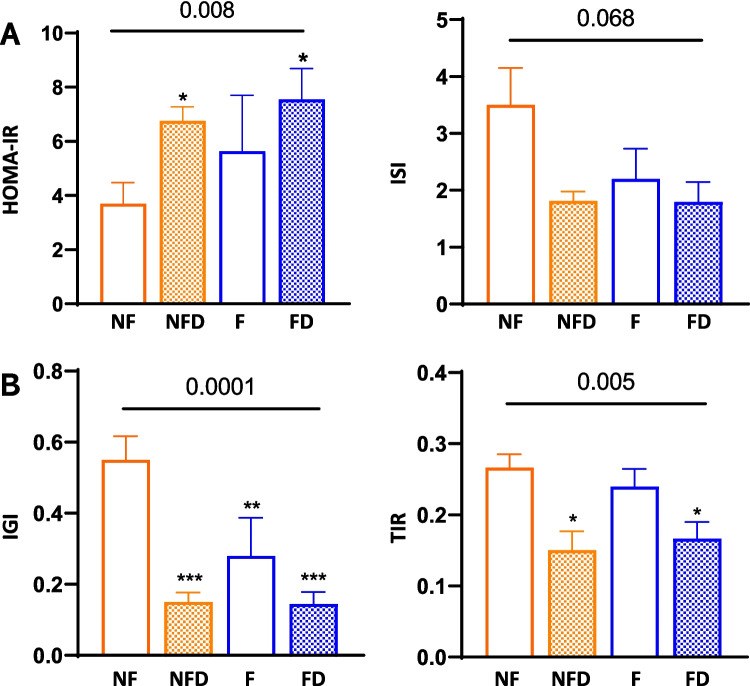
Indices of insulin sensitivity and secretion in patients with MASLD. Panel A, HOMA-IR and insulin sensitivity index (ISI). Panel B, insulinogenic index (IGI) and total insulin response (TIR) during MTT. Group differences were analysed by one-way ANOVA (numeric p-value for Groups). The pairwise comparisons vs. reference group (NF) are Tukey post-hoc tests (* < 0.05, ** < 0.01, *** < 0.001, and **** < 0.0001). Abbreviations: NF: no liver fibrosis or T2D; NFD: no liver fibrosis with T2D; F: liver fibrosis without T2D; and FD: both liver fibrosis and T2D

Altered glucose homeostasis was reflected in insulin resistance indices. Specifically, HOMA-IR values were significantly elevated in the NFD, F, and FD groups (1.8-, 1.5-, and 2.0-fold, respectively) compared to the NF group (*p* = 0.008), alongside a trend toward lower ISI values (*p* = 0.068; Fig. 2A). Insulinogenic Index (IGI) was significantly reduced in all groups compared to the NF group (*p* = 0.0001), with the F group exhibiting a ~ 50% decrease relative to NF (*p* = 0.002). A comparable pattern emerged when assessing the total insulin response (TIR) during the MTT using the insulin-to-glucose AUC ratio (*p* = 0.005), though values for the F group remained similar to those of the NF group (Fig. 2B). These results suggest a potential relationship between hepatic fibrosis and changes in glucose metabolism and beta-cell function, regardless of T2D status.

### T2D disrupts postprandial glucagon response in MASLD independent of fibrosis

**Fig. 3:**
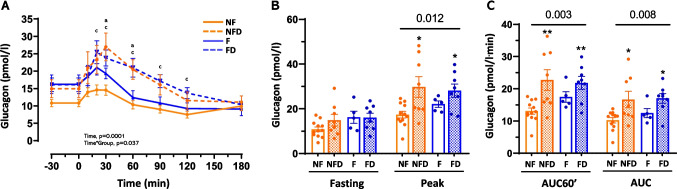
Glucagon levels and dynamics during MTT in patients with MASLD. Panel A shows glucagon profiles, panel B fasting and peak levels, and panel C mean levels for the first 60 min and the entire test (AUC60′ and AUC, respectively) in response to MTT. Profiles were analysed by repeated-measures ANOVA. An one-way ANOVA was performed for each time-point. The Tukey post-hoc test is indicated as (a, b, or c) for the comparison of NFD, F, and FD groups with the NF group, indicating a *p* < 0.05, respectively. Panel B and C were analysed by one-way ANOVA (numeric p-values for groups). Pairwise comparisons are Tukey’s post-hoc tests (* < 0.05, ** < 0.01, *** < 0.001, and **** < 0.0001). Abbreviations: NF: no liver fibrosis or T2D; NFD: no liver fibrosis with T2D; F: liver fibrosis without T2D; and FD: both liver fibrosis and T2D

Physiological responses to meal ingestion typically involve insulin secretion and suppression of glucagon release. In this study, people with T2D (NFD and FD groups) failed to exhibit the expected postprandial glucagon suppression during the MTT, instead showing elevated glucagon levels regardless of fibrosis status (*p* = 0.003; Fig. 3A-C; Table 2). Peak glucagon values and AUC measurements (AUC60’ and total AUC) were significantly higher in T2D groups compared to NF (*p* < 0.05; Fig. 3B-C; Table 2), while fasting glucagon levels remained similar across groups. Individuals with fibrosis but without T2D (F group) displayed an intermediate glucagon response, falling between the NF and T2D groups (*p* = 0.037; Fig. 3A; Table 2). These findings suggest that T2D disrupts meal-induced glucagon suppression in MASLD, while fibrosis alone partially influences glucagon dynamics without the pronounced dysregulation observed in T2D.

**Table 2:** Fasting and post-prandial hormone responses

variables	NF	NFD	F	FD	ANOVA
**Glucagon**					
Fasting (pmol/l)	10.8 ± 3.6	14.9 ± 7.4	16.2 ± 6.0	16.1 ± 5.6	0.132
Peak (pmol/l)	17.1 [7.8]	25.7 [24.2]*	22.8 [6.1]	28.0 [16.0]*	0.012
AUC60' (pmol/l*min)	12.7 [4.9]	20.6 [15.4]**	16.1 [5.9]	22.4 [9.2] **	0.003
iAUC60' (pmol/l*min)	2.8 [2.8]	5.9 [9.1] *	2.0 [6.7]	3.6 [10.7]	0.026
AUC (pmol/l*min)	10.2 ± 2.9	16.7 ± 7.3*	12.5 ± 3.2	17.0 ± 4.3*	0.008
iAUC (pmol/l*min)	−0.6 ± 2.0	1.8 ± 5.4	−3.7 ± 3.3	1.0 ± 5.7	0.143
**GLP-1**					
Fasting (pmol/l)	25.5 ± 9.9	38.8 ± 10.7	41.7 ± 12.6	48.9 ± 21.2**	0.008
Peak (pmol/l)	41.5 ± 10.1	59.4 ± 16.6	69.7 ± 29.9*	76.0 ± 19.5**	0.002
AUC60' (pmol/l*min)	34.0 ± 10.3	49.4 ± 14.0	60.4 ± 23.3*	63.6 ± 22.7**	0.004
iAUC60' (pmol/l*min)	8.6 ± 5.5	10.6 ± 8.4	18.7 ± 15.1	14.7 ± 5.7	0.128
AUC (pmol/l*min)	29.9 ± 7.8	41.8 ± 12.0	47.2 ± 18.1	56.3 ± 19.8**	0.004
iAUC (pmol/l*min)	4.7 [6.5]	3.2 [12.5]	9.4 [15.8]	5.9 [9.4]	0.719
**GLP-2**					
Fasting (pg/ml)	3.2 [1.1]	4.3 [1.0]	5.6 [2.6]	5.2 [4.5]*	0.016
Peak (pg/ml)	4.1 [1.4]	5.1 [2.0]	5.5 [2.8]	6.4 [4.4] **	0.007
AUC60' (pg/ml*min)	4.6 [1.6]	5.6 [1.8]	6.2 [3.1]	7.0 [5.8] **	0.008
iAUC60' (pg/ml*min)	1.1 [0.5]	1.4 [1.3]	1.5 [0.9]	1.8 [1.3] **	0.009
AUC (pg/ml*min)	3.5 [1.0]	4.3 [1.6]	4.9 [2.9]	5.5 [3.8] **	0.006
iAUC (pg/ml*min)	0.13 ± 0.46	0.27 ± 0.36	−0.19 ± 0.41	0.36 ± 0.55	0.274
**GIP**					
Fasting (pg/ml)	68.9 ± 29.8	126.5 ± 32.0**	62.4 ± 19.1	103.3 ± 34.0	0.001
Peak (pg/ml)	658.9 ± 274.7	802.6 ± 371.5	825.8 ± 398.1	653.5 ± 227.0	0.600
AUC60' (pg/ml*min)	414.3 [324.2]	610.5 [499.1]	545.3 [479.7]	484.3 [342.1]	0.500
iAUC60' (pg/ml*min)	383.0 ± 178.5	478.4 ± 178.5	522.7 ± 255.2	388.5 ± 183.4	0.562
AUC (pg/ml*min)	451.5 ± 254.7	572.3 ± 223.6	532.5 ± 221.5	435.0 ± 164.6	0.388
iAUC (pg/ml*min)	382.6 ± 127.5	445.8 ± 225.8	470 ± 212.7	331.7 ± 167.2	0.489

Data are mean ± SD or median [IQR] depending on data distribution. Fasting and peak are absolute values while the areas under the curve (AUC60’ and AUC) and the incremental areas under the curves (iAUC60’ and iAUC) are shown as the mean value calculated from the relative period (60 or 180 min). Group differences were assessed by one-way ANOVA with Tukey’s post-hoc test for pairwise comparisons. The symbol (*) shows the comparison between the reference group and the NFD, F, and FD groups: *p < 0.05, **p < 0.01, ***p < 0.001, and ****p < 0.0001

### Patients with MASLD with liver fibrosis and T2D show impaired GLP-1 responses but preserved GIP responses

**Fig. 4:**
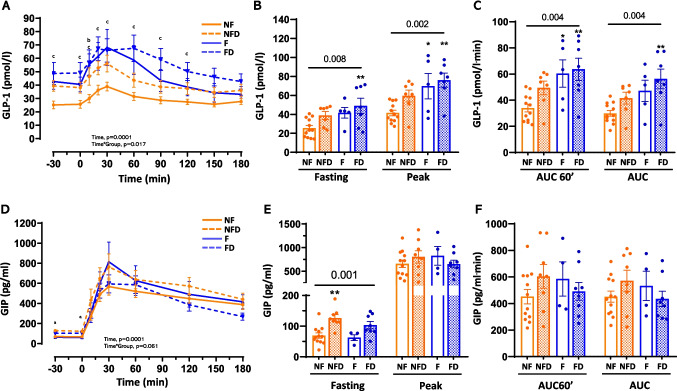
GLP-1 and GIP levels and dynamics during MTT in patients with MASLD. Panels A and D show GLP-1 and GIP profiles, respectively, in response to the MTT. Panels B and E show the fasting and peak GLP-1 and GIP levels, and panels C and F show the mean GLP-1 and GIP levels for the first 60 min, and for the entire period of the test (AUC60’ and AUC, respectively), respectively. The profiles in A and C were analysed by ANOVA for repeated measures while an one-way ANOVA was performed for each time-point. The Tukey post-hoc test is indicated as (a, b, or c) for the comparison of NFD, F, and FD groups with the NF group, indicating a p < 0.05, respectively. Variables in panels B, C, E, and F were analysed by one-way ANOVA (numeric p-value for Groups). The pairwise comparisons vs. reference group (NF) are Tukey post-hoc tests (* < 0.05, ** < 0.01, *** < 0.001, and **** < 0.0001). Abbreviations: NF: no liver fibrosis or T2D; NFD: no liver fibrosis with T2D; F: liver fibrosis without T2D; and FD: both liver fibrosis and T2D

Liver fibrosis (F group) and T2D (NFD and FD groups) were associated with elevated fasting GLP-1 levels and enhanced postprandial responses during the MTT (*p* = 0.008 and *p*_int_ = 0.017; Fig. 4A, B). Fasting GLP-1 levels showed a trend toward an increase in the F group, almost doubling compared with baseline values (25.5 ± 9.9 vs. 41.7 ± 12.6 pmol/L; *p* = 0.126), while peak GLP-1 levels were significantly higher compared to NF (41.5 ± 10.1 vs. 69.7 ± 29.9 pmol/L; *p* = 0.03; Fig. 4B; Table 2). Early GLP-1 AUC (AUC60’) was also elevated in fibrosis groups, irrespective of T2D status (*p* < 0.05; Fig. 4C; Table 2).

In contrast, GIP responses remained relatively stable across groups (*p*_int_ = 0.061; Fig. 4D). Fasting GIP levels differed significantly between groups (*p* = 0.001), with the NFD group showing higher levels compared to NF (*p* = 0.002). The non-T2D groups (NF and F) exhibited similar, lower fasting GIP levels, and no other significant differences in postprandial GIP dynamics were observed (Fig. 4D-F; Table 2). These findings reveal distinct secretion patterns of GLP-1 and GIP in MASLD: GLP-1 responses are markedly altered in fibrosis and T2D, while GIP secretion remains largely unaffected across disease stages.

### Combined liver fibrosis and T2D significantly enhance basal and postprandial GLP-2 secretion

**Fig. 5:**
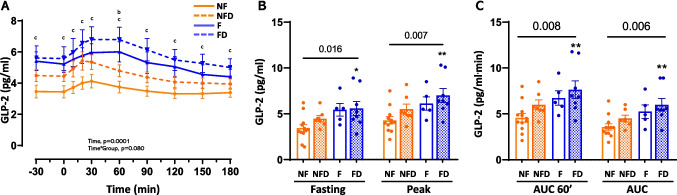
GLP-2 levels and dynamics during MTT in patients with MASLD. Panel A shows GLP-2 profile in response to the MTT. Panel B shows the fasting and peak GLP-2 levels, and panel C shows the mean GLP-2 levels for the first 60 min and for the entire period of the test (AUC60’ and AUC, respectively). The profiles were analysed by ANOVA for repeated measures. An one-way ANOVA was performed for each time-point. The Tukey post-hoc test is indicated as (a, b, or c) for the comparison of NFD, F, and FD groups with the NF group, indicating a p < 0.05, respectively. Variables in panel B were analysed by one-way ANOVA (numeric p-value for Groups). The pairwise comparisons vs. reference group (NF) are Tukey post-hoc tests (* < 0.05, ** < 0.01, *** < 0.001, and **** < 0.0001). Abbreviations: NF: no liver fibrosis or T2D; NFD: no liver fibrosis with T2D; F: liver fibrosis without T2D; and FD: both liver fibrosis and T2D

Liver fibrosis and T2D altered the GLP-2 response to the MTT (Fig. 5A), mirroring the pattern observed for GLP-1 (Fig. 4A). Fasting GLP-2 levels were significantly higher in the FD group compared to NF (3.2 [1.1] vs. 5.2 [4.5] pmol/L; *p* = 0.025), as were peak GLP-2 levels (4.1 [1.4] vs. 6.4 [4.4] pmol/L; *p* = 0.005; Fig. 5B; Table 2). Similarly, AUC60’ and total AUC values for GLP-2 were 1.5- and 1.6-fold higher in the FD group than in NF (*p* = 0.007 and *p* = 0.005, respectively). These findings suggest that, in MASLD, T2D, and fibrosis can influence basal and postprandial GLP-2 levels, with the observed changes most pronounced when both conditions are present concurrently.

### Liver fibrosis as a determinant of secretory patterns of GLP-1 and GLP-2

To identify the main determinants of incretin hormone secretory patterns, we conducted a stepwise regression analysis using MTT-derived indices as dependent variables. Age, FLI, TE, body mass index (BMI), fasting plasma glucose (FPG), or glycated hemoglobin (HbA1c) were included as potential predictor variables (Table 3).

**Table 3:** Stepwise regression models

	**Variable**	**β (standardised)**	**P-value**	**Model R**^**2**^
**Glucagon AUC**	HbA1c	0.381	0.010	**0.145**
**Glucagon iAUC**	HbA1c	0.396	0.030	**0.127**
**Glucagon AUC60’**	FPG	0.372	0.027	**0.205**
	BMI	0.360	0.032	
**Glucagon iAUC60’**	HbA1c	0.450	0.013	**0.202**
**Fasting GLP-1**	ET	0.548	0.0001	**0.504**
	FPG	0.417	0.004	
**GLP-1 peak**	ET	0.675	0.0001	**0.586**
	FPG	0.291	0.027	
**GLP-1 AUC**	ET	0.652	0.0001	**0.618**
	FPG	0.398	0.002	
**GLP-1 AUC60’**	ET	0.636	0.0001	**0.535**
	FPG	0.322	0.019	
**GLP-1 iAUC60’**	ET	0.465	0.008	**0.216**
**Fasting GLP-2**	BMI	0.631	0.0001	**0.398**
**GLP-2 peak**	BMI	0.464	0.004	**0.451**
	ET	0.349	0.027	
**GLP-2 AUC**	BMI	0.437	0.007	**0.454**
	ET	0.382	0.016	
**GLP-2 AUC60’**	BMI	0.516	0.001	**0.479**
	ET	0.314	0.04	
**GLP-2 iAUC60’**	BMI	0.481	0.006	**0.232**
**Fasting GIP**	FPG	0.739	0.0001	**0.546**

Variables included in the regression models were age, FLI, ET, BMI and FPG or HbA_1c_ values. Fasting and peak are absolute values while the areas under the curve (AUC60’ and AUC) and the incremental areas under the curves (iAUC60’ and iAUC) were computed as the mean value calculated from the relative period (60 or 180 min)

Our regression models revealed that liver fibrosis, as measured by TE values, emerged as the strongest predictor of GLP-1 secretory patterns. TE demonstrated robust associations with fasting GLP-1 levels (β = 0.548, *p* = 0.0001, R^2^ = 0.504), GLP-1 peak concentrations (β = 0.675, *p* = 0.0001, R^2^ = 0.586), GLP-1 total area under the curve (AUC; β = 0.652, *p* = 0.0001, R^2^ = 0.618), and GLP-1 AUC60' (β = 0.636, *p* = 0.0001, R^2^ = 0.535). These high R^2^ values indicate that liver fibrosis accounts for a substantial portion of the variability in GLP-1 secretion parameters.

For GLP-2 secretion, our analysis identified a more complex set of determinants. Fasting GLP-2 levels were predominantly predicted by BMI (β = 0.631, *p* = 0.0001, R^2^ = 0.398). The dynamic GLP-2 responses showed significant associations with both BMI and TE, with BMI being the stronger predictor for GLP-2 peak levels (β = 0.464, *p* = 0.004), GLP-2 AUC (β = 0.437, *p* = 0.007, R^2^ = 0.454), and GLP-2 AUC60' (β = 0.516, *p* = 0.001, R^2^ = 0.479). TE also contributed significantly to predicting these measures, with standardized coefficients ranging from 0.314 to 0.362.

These findings highlight the importance of liver fibrosis as a key determinant of incretin hormone secretion patterns, particularly for GLP-1. The statistical strength of TE as a predictor across multiple GLP-1 parameters suggests that liver status may be an important consideration when evaluating incretin hormone dynamics in metabolic-associated steatotic liver disease (MASLD).

## Discussion

This study underscores the influence of liver fibrosis on the regulation of glucagon-related peptide secretion, most notably GLP-1, in individuals diagnosed with MASLD. The observed data indicate that liver fibrosis is the most consistent and significant determinant of circulating GLP-1 levels in both fasting and postprandial conditions. Additionally, the coexistence of type 2 diabetes (T2D) appears to amplify these effects, indicating a potential synergistic interplay between liver fibrosis (liver stiffness) and persistent hyperglycaemia in modulating incretin hormone dynamics. Across all measures, including fasting levels, postprandial peak responses during the MTT, and the AUC, GLP-1 concentrations were significantly elevated in individuals with liver fibrosis. These associations remained statistically significant after adjusting for key metabolic variables, including body mass index (BMI), waist circumference, and fasting plasma glucose (FPG), supporting the notion that liver fibrosis contributes independently to altered incretin dynamics. Although the mechanisms underlying these associations are yet to be fully elucidated, it is conceivable that fibrosis-related alterations in hepatic structure and function may influence GLP-1 secretion or clearance. Potential pathways could include changes in hepatic extraction of incretin hormones [24], disruptions in bile acid metabolism [25], or modifications in gut microbiota composition, which have been linked to the regulation of enteroendocrine function [26, 27]. Nevertheless, these hypotheses remain speculative and should be interpreted cautiously, as the present study was not designed to investigate causality or delineate mechanistic pathways.

The presence of T2D further modifies incretin profiles. People with both liver fibrosis and T2D exhibited the highest levels of GLP-1 under both fasting and stimulated conditions, suggesting an interaction wherein chronic hyperglycemia amplifies the hepatic contribution to incretin dysregulation. Persistent hyperglycemia may lead to desensitization of GLP-1 receptors, resulting in compensatory upregulation of hormone production [28]. This interpretation is consistent with existing literature supporting the reciprocal exacerbation of MASLD and T2D, in which metabolic and hepatic abnormalities reinforce one another in a self-perpetuating cycle [3–6].

GLP-2 exhibited a similar, albeit less pronounced, pattern to GLP-1. While fasting GLP-2 concentrations were primarily associated with BMI, postprandial levels were influenced by both BMI and liver stiffness. Given the known role of GLP-2 in maintaining intestinal barrier integrity and regulating the gut-liver axis [17], its elevation in the context of liver fibrosis may represent a physiological adaptation to counteract the effects of increased intestinal permeability and inflammation [18, 29, 30], However, as with GLP-1, interpreting these associations remains hypothetical and requires further mechanistic investigation to establish causality or physiological relevance.

Our findings also revealed distinct patterns of glucagon regulation. While glucagon secretion was markedly elevated and dysregulated in individuals with T2D, patients with liver fibrosis in the absence of diabetes exhibited intermediate glucagon levels. This observation suggests that liver fibrosis alone may partially impair glucagon regulation, though the full extent of dysregulation observed in T2D likely reflects additional metabolic and pancreatic dysfunction. Hyperglucagonemia, in turn, contributes to increased hepatic glucose output and may further impair glycemic control, particularly in individuals with coexisting MASLD and T2D [9, 12].

These findings may have clinical implications, particularly potential therapeutic strategies targeting the incretin axis. GLP-1 receptor agonists (GLP-1RAs) have shown substantial efficacy in managing T2D and MASLD, with clinical trials demonstrating reductions in hepatic fat and improvements in fibrosis-related markers [31]. The elevated endogenous GLP-1 levels observed in patients with advanced fibrosis may suggest a state of relative GLP-1 resistance [15, 27, 28], potentially necessitating pharmacological doses to achieve therapeutic benefit. This notion may partly explain the preserved efficacy of GLP-1RAs despite elevated baseline hormone concentration in a similar patient population [32]. Although this study focused on incretin hormones, other metabolic and inflammatory mediators such as adipokines, hepatokines, and cytokines can also influence their secretion. These factors may either stimulate or suppress incretin release depending on the metabolic and inflammatory state. Therefore, the observed hormonal changes may not be unique to MASLD but could also occur in other metabolic or liver diseases characterized by inflammation and insulin resistance [33–36].

## Strengths and limitations

This study offers several strengths. It provides a comprehensive assessment of glucagon-related peptides (GLP-1, GLP-2, GIP, and glucagon) across various stages of MASLD and T2D, employing a standardized MTT protocol. Furthermore, the use of stepwise multivariable regression models enabled the identification of independent predictors of hormone levels while accounting for potential confounders such as BMI and FPG.

Nonetheless, some limitations should be acknowledged. Hepatic steatosis and liver stiffness were assessed using the FLI and TE, respectively. Although these are non-invasive and widely accepted methods, they lack histological resolution and should be interpreted with this methodological consideration in mind. We are also aware that our results should be validated in independent cohorts with biopsy-confirmed MASLD and liver fibrosis, as liver biopsy remains the gold standard for evaluating the full histological spectrum of the disease. Moreover, this is a single-center study, so that the findings may be closely linked to the specific characteristics of the local population, as well as the procedures and expertise of that particular center. The absence of a healthy control group precludes definitive conclusions regarding the specificity of hormonal alterations in MASLD relative to generalized metabolic dysfunction. Additionally, subgroup analyses, particularly those involving participants with fibrosis without T2D, were limited by small sample sizes, which may have reduced statistical power.

## Conclusions

In conclusion, liver fibrosis emerges as a key determinant of incretin hormone secretion patterns in MASLD, with forceful associations observed for GLP-1. T2D intensifies these alterations, suggesting a complex interplay between hepatic fibrosis and metabolic dysfunction in modulating enteroendocrine responses.

The interaction between T2D and liver fibrosis in shaping incretin responses underscores the need for early, integrated intervention strategies. Simultaneous targeting of metabolic dysregulation and liver pathology may disrupt the pathogenic interplay between these conditions, thereby improving glycemic control and hepatic outcomes. Recent clinical data support this approach, indicating that early metabolic correction may decelerate or even reverse fibrosis progression in patients with MASLD [31].

## Data Availability

The data that support the findings of this study are available from the corresponding author, Sonia Fernández-Veledo and Joan Vendrell, upon reasonable request.

## Abbreviations

FLI: Fatty liver index
FPG: Fasting plasma glucose
GLP-1: Glucagon-like peptide-1
GLP-2: Glucagon-like peptide-2
GIP: Glucose-dependent insulinotropic polypeptide
ISI: Insulin sensitivity index
IGI: Insulinogenic index
TE: Transient elastography
MTT: Meal tolerance test
MASLD: Metabolically associated steatotic liver disease
MASH: Metabolic dysfunction-associated steatohepatitis
TIR: Total insulin response
T2D: Type 2 diabetes
